# Synthesis and investigation on microstructural, mechanical features of mesoporous hardystonite/reduced graphene oxide nanocomposite for medical applications

**DOI:** 10.3389/fbioe.2023.1073435

**Published:** 2023-03-13

**Authors:** Iman Bagherpour, Amirhossein Yaghtin, Seyed Morteza Naghib, Fatemeh Molaabasi

**Affiliations:** ^1^ Department of Materials Science and Engineering, College of Engineering No.2, Islamic Azad University, Shiraz branch, Iran; ^2^ Nanotechnology Department, School of Advanced Technologies, Iran University of Science and Technology (IUST), Tehran, Iran; ^3^ Biomaterials and Tissue Engineering Research Group, Department of Interdisciplinary Technologies, Breast Cancer Research Center, Motamed Cancer Institute, ACECR, Tehran, Iran

**Keywords:** hardystonite, reduced graphene oxide, mechanical properties, biocompatibility, nanoparticles

## Abstract

The use of hardystonite (Ca_2_ZnSi_2_O_7_, HT)-based composites could be one the main strategies to improve mechanical properties closing to natural bone. However, there are a few reports in this regard. Recent findings indicate that graphene is a promising biocompatible additive in ceramic-based composite. Here, we propose a simple approach for the synthesis of porous nano- and microstructured hardystonite/reduced graphene oxide (HT/RGO) composite using a sol-gel method followed by ultrasonic and hydrothermal processes. Integrating GO to the pure HT increased the bending strength and toughness values about 27.59% and 34.33%, respectively. It also allowed the increment of compressive strength and compressive modulus by about 8.18% and 86%, respectively, and improvement in the fracture toughness about 11.8 times compared to pure HT. The formation of HT/RGO nanocomposites with different RGO weight percentages ranging from 0 to 5.0 has been investigated by scanning electron microscopy (SEM) and X-ray diffraction and the efficient incorporation of GO nanosheets into HT nanocomposite as well as the mesoporous structural properties were also confirmed by Raman, FTIR and BET analyses. The cell viability of HT/RGO composite scaffolds was assayed by methyl thiazole tetrazolium (MTT) test *in vitro*. In this regard, the alkaline phosphatase (ALP) activity and the proliferation rate of mouse osteoblastic cells (MC3T3-E1) on the HT/1 wt. % RGO composite scaffold enhanced in comparison with the pure HT ceramic. The adhesion of osteoblastic cells on the 1% wt. HT/RGO scaffold was interesting as well. In addition, the effect of 1% wt. HT/RGO extract on the proliferation of osteoblast human G-292 cells was successfully evaluated and remarkable observations were obtained. All together it can be said that the proposed bioceramic hardystonite/reduced graphene oxide composites can be a promising candidate for designing hard tissue implants.

## Graphical Abstract

**Figure:**
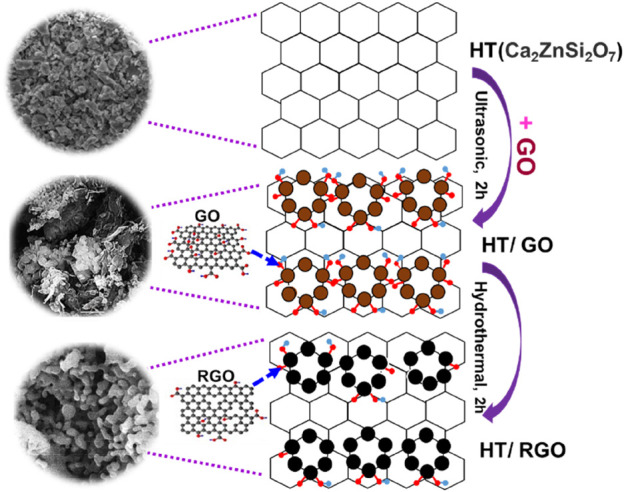

## 1 Introduction

The field study of graphene-based nanocomposites has received a lot of attention over the past decade in various projects, such as biosensing, bioimaging, and tissue engineering. The addition of graphene-based nanofillers with different geometries and structures, such as graphene oxide (GO), reduced graphene oxide (RGO), and graphene nanoplatelets (GNPs), could improve the mechanical and electrical properties of polymer matrix (Ramanathan et al., 2008; Zhou et al., 2012; Castelaín et al., 2013), ceramic and bioceramic composites, such as hydroxyapatite (HA) (Liu et al., 2013a; Zhang et al., 2013), Si_3_N_4_ (Walker et al., 2011; Ramirez et al., 2014), Al_2_O_3_ (Liu et al., 2013b), zirconia/alumina composites (Liu et al., 2012), and calcium phosphate composites (Zhao et al., 2013). Watcharotone et al. fabricated an electrical transparent conductor on hydrophilic SiO_x_/silicon and glass substrates using mixing GO nanosheets with the silica solution by a simple sol-gel method followed by chemical reduction, spin-coating, and thermal curing (Watcharotone et al., 2007). The developed films exhibited a favorable electrical conductivity in comparison with thin films of carbon nanotubes (CNTs) in silica. In addition, graphene showed less risk of impurity-induced toxicity compared to CNTs due to the synthesis of graphene in a more pure environment (Liu et al., 2013a). Li et al. (2013) could prepare GO- based nanohydroxyapatite (HA) on pristine and chitosan with enhanced cytocompatibility using spark plasma sintering (SPS). Zhang et al. (2013) synthesized GNP/HA composite to report the improvement of *in vitro* biocompatibility, suitable bone bonding ability, and good deposition of plate-like HA in SBF solution in comparison with pure HA.

Recently, reduced graphene oxide (RGO) has been proposed that can be used as an alternative material for graphene. To produce RGO, chemical treatment, thermal annealing, microwave or various microbial and bacterial methods could be applied to eliminate oxygen functional groups from GO surface (He et al., 2011; Hu et al., 2013; Priyadarsini et al., 2018). Recently Agarwal et al. (2010) demonstrated the biocompatibility of RGO for 3 cell lines including human oligodendroglia cell line HOG, human fetal osteoblast cell line hFOB, and rat pheochromocytoma cell line PC12 . In addition, RGO has been utilized in osteogenic stem cells to research on myogenesis (Farzin et al., 2017; Li et al., 2017; No et al., 2020), epithelial genesis (Hamvar et al., 2020), neurogenesis (Raya et al., 2021), and cardiomyogenesis (Lin et al., 2007; Priyadarsini et al., 2018; Kianfar, 2021). Liu et al. reported the incorporation of RGO in HA for the case of load-bearing orthopedic implants which not only has shown biocompatibility on hFOB cells, but also increases fracture toughness as compared to the pure HA (Liu et al., 2013a). Moreover, RGO/HAp graft increases considerably higher bone density (52%) than untreated control (17%) as well as HAp (26%) alone (Lee et al., 2015). Mehrali et al. found that the RGO reinforcement in calcium silicate (CaSiO_3_, CS) using a hydrothermal method accompanied with hot isostatic pressing (HIP) could significantly improve the fracture toughness of CS/RGO composites. Nosrati et al. (2019) has synthesized a hybrid HA/RGO by hydrogen gas injection process into a hydrothermal autoclave. As prepared hybrid material showed high crystallinity as well as an increased mechanical property. However, to the best of the authors’ knowledge, there are no reports on the biological and mechanical properties by ceramic composites containing hardystonite and RGO.

Hardystonite (Ca_2_ZnSi_2_O_7_, HT) with Zn incorporation into calcium silicate ceramic has shown better chemical stability and more mechanical strength compared to other CS ceramics such as CaMgSi_2_O_6_ and Ca_2_ SiO_4_ (Diba et al., 2014; Gheisari et al., 2015; Bagherpour et al., 2018). Moreover, HT ceramics can promote the attachment, proliferation and differentiation of human osteoblast-like cells (hFOB), improve the apatite formation, and enhances the alkaline phosphatase activity, making it an interesting bio-based candidate for hard tissue repair (Srinath et al., 2020). However, the mechanical and biological performance of HT can be further improved by incorporating second reinforcements such as other ceramics (HA, CaSiO_3_, Si_3_N_4_) (Long et al., 2008; Zhao et al., 2008; Walker et al., 2011; Liu et al., 2013a; Mehrali et al., 2014; Gheisari et al., 2015), and polymers (chitosan; poly capro lactone (PCL)) (Tang et al., 2012; Caballero et al., 2019). Li et al. demonstrated the potential of the sodium alginate (SA)/HT hydrogel biocomposite as a multifunctional wound dressing to inhibit bacterial growth and promote angiogenesis and wound healing (Li et al., 2017). This was attributed to functions of Ca^2+^, Zn^2+^ and Si ions in interesting bioactivity of hydrogel biocomposite. Moreover, No et al. successfully indicated the positive effect of strontium-hardystonite (Sr-HT) in polyvinyl alcohol/gelatin composite hydrogel (FRH-PG) for tendon graft applications (No et al., 2020). Farzin et al. has developed the multifunctional Fe-doped HT, i.e., 0.15Fe-HT and 0.25Fe-HT, by the sol–gel method with the aim of tissue engineering, drug delivery and hyperthermic applications (Farzin et al., 2017). Khanna et al. (2017) also incorporated HA and HT ceramics in PCL nanofibers and demonstrated clearly better functionality of HT-PCL compared to the HA-PCL for bone regeneration. In addition, Hamvar et al. (2020) proved the synergistic effect of 12.5 wt% of diopside (CaMgSi_2_O_6_), a silicate-based ceramic, in the HT scaffold (HT/Di) by *in vitro* cellular tests. However, few cost-effective HT composites with a favorable combination of biocompatibility and mechanical strength have been reported so far.

The hydrothermal process, known as a low cost, simple, and non-polluting method, is one of the popular methods to synthesize homogeneous CS that could effectively enhance the crystallinity of the bioceramic product (Lin et al., 2007). In addition, sol-gel is an industrial and conventional method for producing ceramic nanostructures and nanocomposites with outstanding advantages including the narrow size distribution of product, the high purity (99.99%) with highly homogeneously composites, and the achievement of uniform structures at low temperatures. The achievement of continuous nano-porosity with large specific surface area without any cracks upon controlled drying of the gel leads as one of the most important issues in this method, which increases the possibility of incorporating secondary materials as well as the rate of compaction of the structure during the sintering process (Kianfar, 2021; Raya et al., 2021). The interesting advantages of mentioned methods causes that sol-gel process in combination with the hydrothermal method could be a promising candidate to create bioceramic nanocomposites because of providing a unique quality of characteristics.

In this study, we report an effective, simple combination of the sol-gel-hydrothermal methods to synthesize porous HT/RGO nanocomposites which are densified using isostatic press. The effect of RGO content on mechanical properties by HT/RGO composites has been systemically evaluated. In addition, *in vitro* experiments including cell proliferation (MTT), cell adhesion, and ALP experiment with respect to the amount of RGO in the matrix were performed to demonstrate the abilities of such developed materials for a perfectly successful biomedical applications in future.

## 2 Materials and methods

### 2.1 Materials

Graphite flakes were purchased from Ashbury, Inc. Sulfuric acid (H_2_SO_4_, 98%), phosphoric acid (H_3_PO_4_, 98%), potassium permanganate (KMnO_4_, 99.9%), hydrogen peroxide (H_2_O_2_, 30%), and hydrochloric acid (HCl, 37%) were purchased from Merck Company. Calcium nitrate tetrahydrate (Ca (NO_3_)_2_·4H_2_O), tetraethyl orthosilicate (TEOS), zinc nitrate hexahydrate (Zn (NO3)2· 6H2O) and sodium metasilicate non-ahydrate (Na_2_SiO_3_·9H_2_O) were purchased from Sigma Aldrich Company. All aqueous solutions were prepared with double-distilled water (DI).

### 2.2 Instrumentation

X-ray diffraction (XRD) device (Bruker, model D8 Advance) was used to confirm the ceramic crystalline structure and phase analysis. Scanning electron microscopy (SEM) (TESCAN, model Vega-3) was employed to investigate the morphology and size of nanostructured particles. Energy-dispersive X-ray spectroscopy (EDX) using the EDX-System (TESCAN, VEGA3) Czech Republic, instrument was used to detect the formed phases according to the previously reported protocol (Bagherpour et al., 2018). Raman analysis (Raman Microscope, TakRam N1-541T Teksan Co,Iran) was applied to identify the existence and property of the GO in nano-/micro hexahydrate bioceramic. FTIR test was employed with IR spectrometer (8500S SHIMADZU) to characterize of functional groups. The BET measurements were carried out on a Micrometritics ASAP2020 system (ASAP 2020) to analyze the surface area of developed HT composite from N2 adsorption and desorption. Moreover, the pore size distribution was obtained from the N2 isotherms based on BJH method.

### 2.3 Preparation of hardystonite powder

Hardystonite (HT) powder was prepared using TEOS, calcium nitrate tetrahydrate, zinc nitrate hexahydrate as reagents *via* sol-gel process. In summary, TEOS was mixed with 1 M HNO_3_ solution and hydrolyzed by shaking for 30 min. Then, calcium nitrate tetrahydrate and zinc nitrate hexahydrate were added to the solution. The reactant agitation was continued at room temperature for 5 h. After that, the solution above was held at 60°C for 24 h and then dried at 120°C for 48 h to yield the dried gel. The obtained dried gel was milled and sieved, and finally transferred to a corundum furnace to calcinate at 1300°C for 3 h resulting nanostructured HT (Bagherpour et al., 2018).

### 2.4 Synthesis of graphene oxide

GO was synthesized according to the Hummer’s method with some modification using the exfoliation of natural graphite. Briefly, 3.0 g of natural graphite powder together with a 20.0 g of potassium permanganate (KMnO_4_) were added and dispersed in 400 ml of acid (HNO_3_: H_2_SO_4_ = 1:9) and stirred for 12 h at 60°C. Then, 10 ml of 30% H_2_O_2_ was slowly spiked in the obtained mixture and then stirred for 60 min resulting in a bright yellow color. Then, the mixture was filtered by a nylon film and washed with double distilled water twice (200 ml). Ultimately, the solid was washed with double distilled water to reach a neutral pH. Finally, the resulting product was dried under vacuum at 60°C and stored in refrigerator before use (Shamsipur et al., 2019).

### 2.5 Synthesis of hardystonite/reduced graphene oxide composite

HT−RGO composite powders with various GO contents (0, 0.5, 1.0, 3.0, and 5.0 wt %) were produced. Specifically, the mixture of GO and HT powders (600 mg) were dispersed into 100 ml ethanol followed by ultrasonically treating for 2 h to ensure its homogeneous distribution. After that, the pristine GO platelets were well exfoliated consistent with previous studies (Li et al., 2014). The pH value of obtained suspensions was adjusted to three to four by HNO_3_ and NH_3_.H_2_O (Li et al., 2014). In the next stage, the above-mentioned solution was held at 60°C for 24 h to obtain HT/GO nanocomposites. It has been reported that thermal treatment is one of the most simple and effective deoxygenating approaches for reducing GO to graphene (Jeong et al., 2009; Chen et al., 2012; Li et al., 2014). To evaluate *in vitro* biocompatibility, HT/RGO composite discs of 6 mmØ × 1.5 mm in size were uniaxially pressed at 10 MPa followed by isostatic pressing at 20 MPa and sintering at 1375°C for 2 h to generate RGO (Tang et al., 2012). Finally, HT/RGO containing various GO contents before and after hydrothermal treatment were characterized using XRD patterns, SEM images, EDX and Raman analysis.

### 2.6 Cell attachment, proliferation and ALP assay

The osteoblastic MC3T3-E1 cell line from the National Cell Bank of Iran (NCBI) was cultured in Duelbacco’s Minimum Eagle’s Medium (DMEM) supplemented with 2 mmol L^−1^ glutamine, 10% fetal bovine serum (FBS), 100 units of potassium penicillin and 100 μg ml^−1^ of streptomycin sulfate. The flask was maintained in 100% atmospheric humidity and 5% CO_2_ incubator at 37°C. When the cells reached the confluence stage, they were harvested by trypsinization and added a fresh culture medium to create a cell suspension. The 3-(4, 5-dimethylthiazol-2-yl)-2, 5-diphenyltetrazolium-bromide (MTT, Aldrich) assay was used to evaluate cell viability after seeding on the scaffolds. Scaffolds were sterilized using conventional autoclave protocol. Furthermore, scaffolds were transferred into the agarose treated 12-well plate and soaked in 1 ml medium for 1 h before seeding. The MC3T3-E1 (5 × 10^4^) cells were seeded on the surface of the scaffolds. The plate was transferred into a cell culture incubator until the MTT treatment. Hardystonite without graphene was considered as control, and the culture medium of wells was replaced with a fresh medium after 2 days. For each composite, experiments were run in triplicate. After culturing the cell on the scaffolds, plats were incubated for 1, 3, and 5 days to study the effect of different incubation times on the cell viability. At each time point, 200 µL of MTT solution (5 mg/ml) was added to each cell culture well (media volume: 1000 μL). After incubation of plate for 4 h at 37°C, the previous solution was slowly removed and followed by 500 μL of DMSO solution added to each well. The cell culture plates were returned to the incubator for 1 h. The absorbance was measured at 570 nm using a BioTek plate reader and data was reported as cell viability (Askari et al., 2021).

ALP is one of the most factors that should be measured in bone tissue engineering. After measuring the viability of cells seeded on the graphene-reinforced hardystonite scaffolds at different incubation times, we selected the pure hardystonite, 0.5, 1, and 3 wt% graphene scaffolds for measuring ALP at 1, 3, and 5 days. The MC3T3-E1 cells were cultured on the surface of selected scaffolds (100 × 10^3^ cell per well) and incubated in a cell culture incubator. According to the manufacturing protocol, ALP Kit (Pars Azmun, Iran) was utilized for the experiment. Absorbance of wells was recorded using 405 nm plate reader (Askari et al., 2021).

To investigate the adhesion and morphology of MC3T3-E1 cells on the developed scaffold, a scanning electron microscopy (SEM) was applied to observe the cell adhesion. For this reason, the cells were seed on the surface of 1 wt% graphene-hardystonite tablet as the obtained optimum scaffold and incubated for 3 days. After that the cells were fixed with 4% glutaraldehyde for 2 h, followed by wash in PBS (0.1 M) and dry at RT (27°C) for SEM images (Askari et al., 2021).

To evaluate biocompatibility of powder extraction, the optimum 1 wt. % graphene-hardystonite sample was selected and extracted according to the ISO 1993–5 protocol. At first, 0.1 g of each powder was placed in culture medium (1 ml) and then kept at 37°C for 3, 5, and 7 days for biocompatibility and proliferation evaluation. Notably, free culture medium was used as negative control group. The biocompatibility assay was performed on the osteoblast human G-292 cells from the NCBI using MTT assay. Briefly, after seeding and culturing cells in a 96-well microtiter plate, the culture medium was removed and replaced with different volume rations (1/30, 1/15, 1/7.5, 1/5, 1/3.75) of composite powder extract solutions (3-day, 5-day and 7-day samples) and 10 µL FBS. After 1, 3, and 5 days of cell culture, MTT assay was run similar to above-mentioned protocol (Mehrjoo et al., 2015).

### 2.7 Mechanical properties evaluation of HT/RGO nanocomposites

To evaluate mechanical properties, HT/RGO composites of RGO various contents (0, 0.5, 1,3 and 5 wt%) with 45.5 mm × 8.0 mm×3.5 mm in size were prepared by uniaxial pressing at 10 MPa followed by isostatic pressing at 20 MPa and sintering at 1375°C for 2 h (Wu et al., 2005).

## 3 Results and discussion

### 3.1 Synthesis and characterization of HT/RGO composites

**FIGURE 1:**
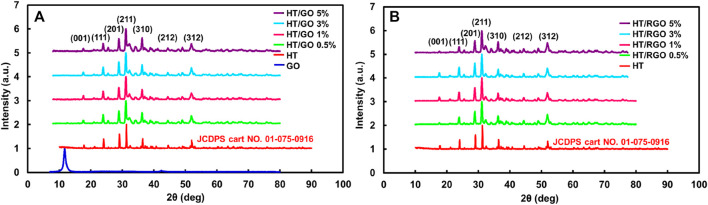
**(A)** XRD patterns of hardystonite bioceramic powders prepared and GO and the composite HT/GO having different weight percentages of GO. **(B)** XRD patterns of the composite HT/RGO.

At first, the powder of HT was analyzed by XRD measurements and then compared with GO and HT/GO composite having different weight percentages of GO (Figure 1). As seen, the XRD pattern of synthesized HT powder confirms the formation of the HT pure phase (standard card no. JCPDS 01-075-0916) of calcium zinc silicate (Ca_2_ZnSi_2_O_7_) with the strongest hardystonite peak at 2θ = 31.3106°, while the tetragonal crystalline structure of HT contained strong diffraction of plan (111), (201), (211), (310), (212) and (312) (Figure 1) (Bagherpour et al., 2018). The XRD spectrum of the GO in Figure 1 accorded with those in other reports and showed a sharp and intense diffraction peak at 2θ = 9.85°, related to the (001) lattice plane consisting with a d-spacing of 0.83 nm. This corresponds to the lamellar structure of GO layers. The peaks in all five HT/GO patterns could be indexed as Ca_2_ZnSi_2_O_7_, since the XRD patterns don’t show the formation of any other phases and also the main diffraction peaks of the HT/GO phase are similar to those of the pure HA phase (Figure 1). Moreover, in the HT/GO composites, the GO could not be detected by XRD for both before and after hydrothermal processing due to its small content (Mehrali et al., 2014). GO sheets in HT composite tablets can be reduced under hydrothermal process, resulting in a very weak (100) peak at 2θ values of 43.4°, corresponding to d-spacing of 0.20 nm. This implies that the GO could be reduced to RGO sheets of composite tablets upon hydrothermal condition due to removing functional groups from the GO surface. Moreover, considering the similarity of XRD patterns between the pure HT and the HT/GO composites before and after the hydrothermal process, suggests preserving the crystal structure of HT in HT/GO composite tablets, so that the main diffraction peaks of the HT phase with RGO are similar to those of the pure HT phase. Moreover, except for (100) peak, no typical diffraction peaks of RGO are recognized in the composite tablets, which can be described by the low amount of RGO and the low diffraction intensity peak (Mehrali et al., 2014).

**FIGURE 2:**
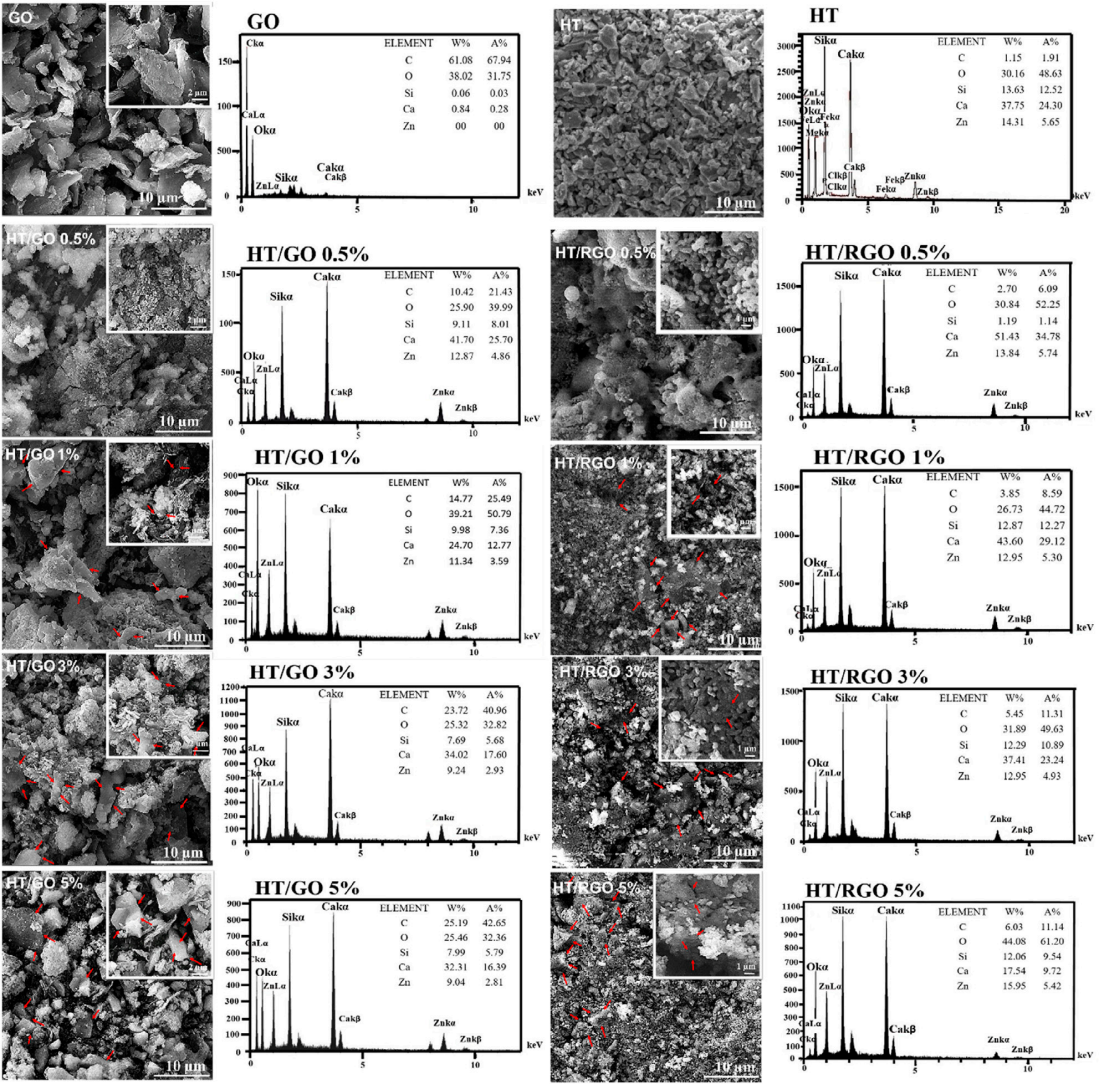
SEM images and EDS patterns of the pure HT, GO and HT composites with different GO and RGO contents.

**FIGURE 3:**
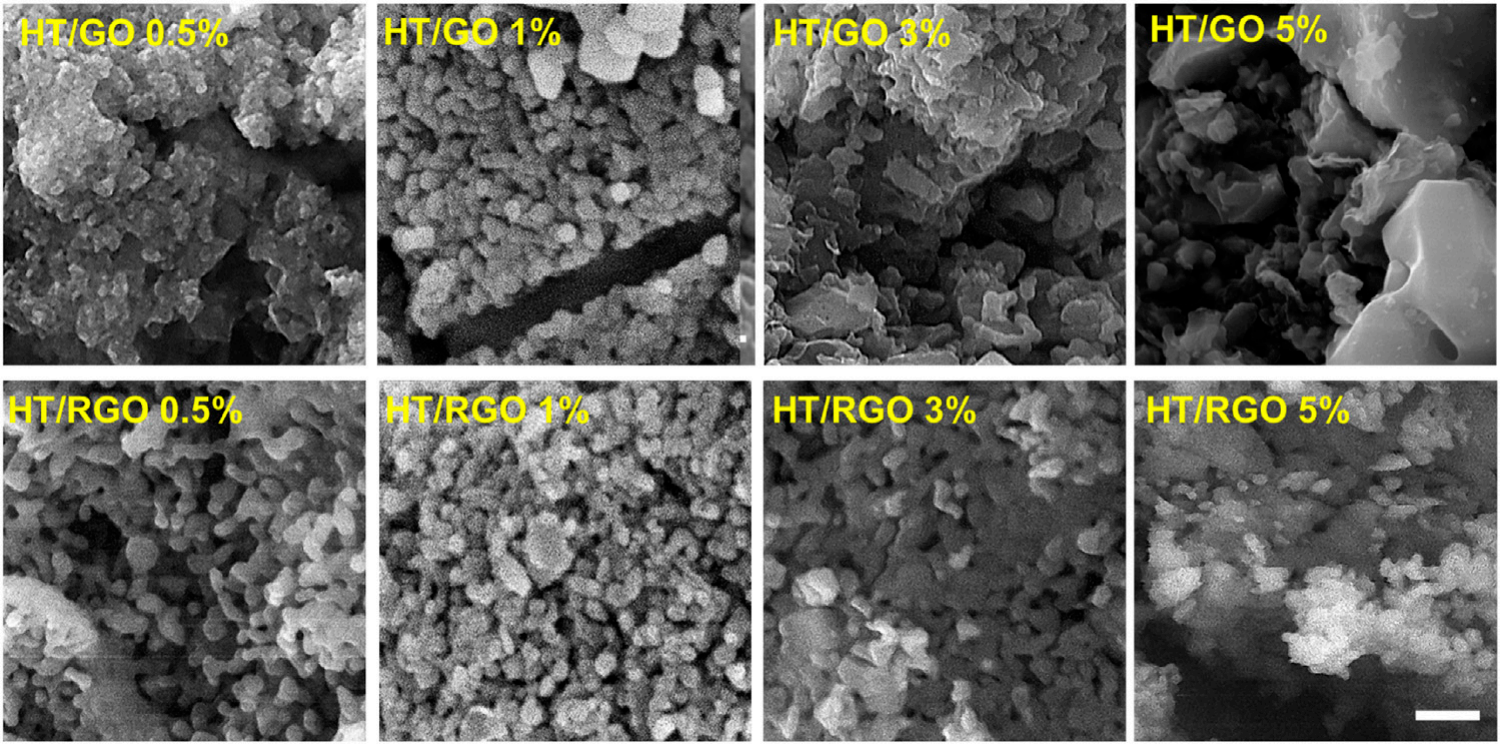
SEM images of the HT composites with different GO and RGO contents. Scale bar ∼1 µm.

The SEM image demonstrated the morphology and particle size of HT ceramics before incorporating with GO. It was clear that most particles are in coarse agglomerates with irregular microstructures and others have nano sizes (≥200 nm) that is consistent with previous studies (Carter and Norton, 2007; Doostmohammadi et al., 2011) (Figure 2). Also, the SEM image of GO shows the agglomeration of wrinkle-like structure with folding/stacking of sheets because of the effective oxygen functionality (Gupta et al., 2017; Jain et al., 2021). As seen, with the addition of GO, the affinity of particles was increased toward the porous regular shapes (pellet-like) (Figures 2, 3). In addition, the SEM images of the HT/GO composite powders (Figure 2) demonstrated the presence of GO sheets in the composite structure as well. As shown in Figure 2, (1% or 5% GO), GO is observed to wrap around HT grains. Really, the high specific surface area of GO causes an increased contact area and thus the bonding strength between GO and HT structures. As seen, the wrapping around the HT pellet-like particles and the dispersion of GO nanosheets in the HT matrix increases with the weight percentage of GO in developed composite. EDS spectra of the powder confirmed the presence of Si, Ca, and Zn basic elements with the definite amounts, indicating the successful synthesized bioceramic HT (Figure 2) (Lee et al., 2015). In addition, the concentration of C increased with an increasing amount of GO in the ceramic (Figure 2), while the Ca/Zn and the Ca/Si molar ratios of the hardystonite in HT/GO composites was found 3.2-3.7 and 4.0-4.6, respectively, close to those of HT (Ca/Zn = 3.5 and Ca/Si = 4.3), suggesting the formation of HT on the HT−GO composites as the amount of GO was increased (Figure 2) (Mehrali et al., 2014). These results are consistent with the XRD results and SEM observations in which the negative effect of incorporation of GO in developed composite is rejected. As depicted in Figures 2, 3, after hydrothermal process, the spherical pellets and spherical grains with more smooth surfaces could be observed and the RGO nanosheets are efficiently incorporated with HT particles. Moreover, except for nano- and microparticles, the HT phase of HT-0.5 wt. % RGO composite and HT-1 wt. % RGO composite appears as nanowires with approximate diameters of 40–70 nm and lengths of several micrometers, similar to the results reported for Xonotlite-1 wt. % RGO composites (Mehrali et al., 2014). In addition, it seems that the structure porosity of HT-0.5 and 1 wt. % RGO composite is more than other composites under hydrothermal treatment, so that the round pellets are more coalesced and connected to each other with increasing the weight percentage of RGO in the composite (Figures 2, 3). This allows a bed of coherent pellet accompanied by a decreasing in the porosity of microstructures.

**FIGURE 4:**
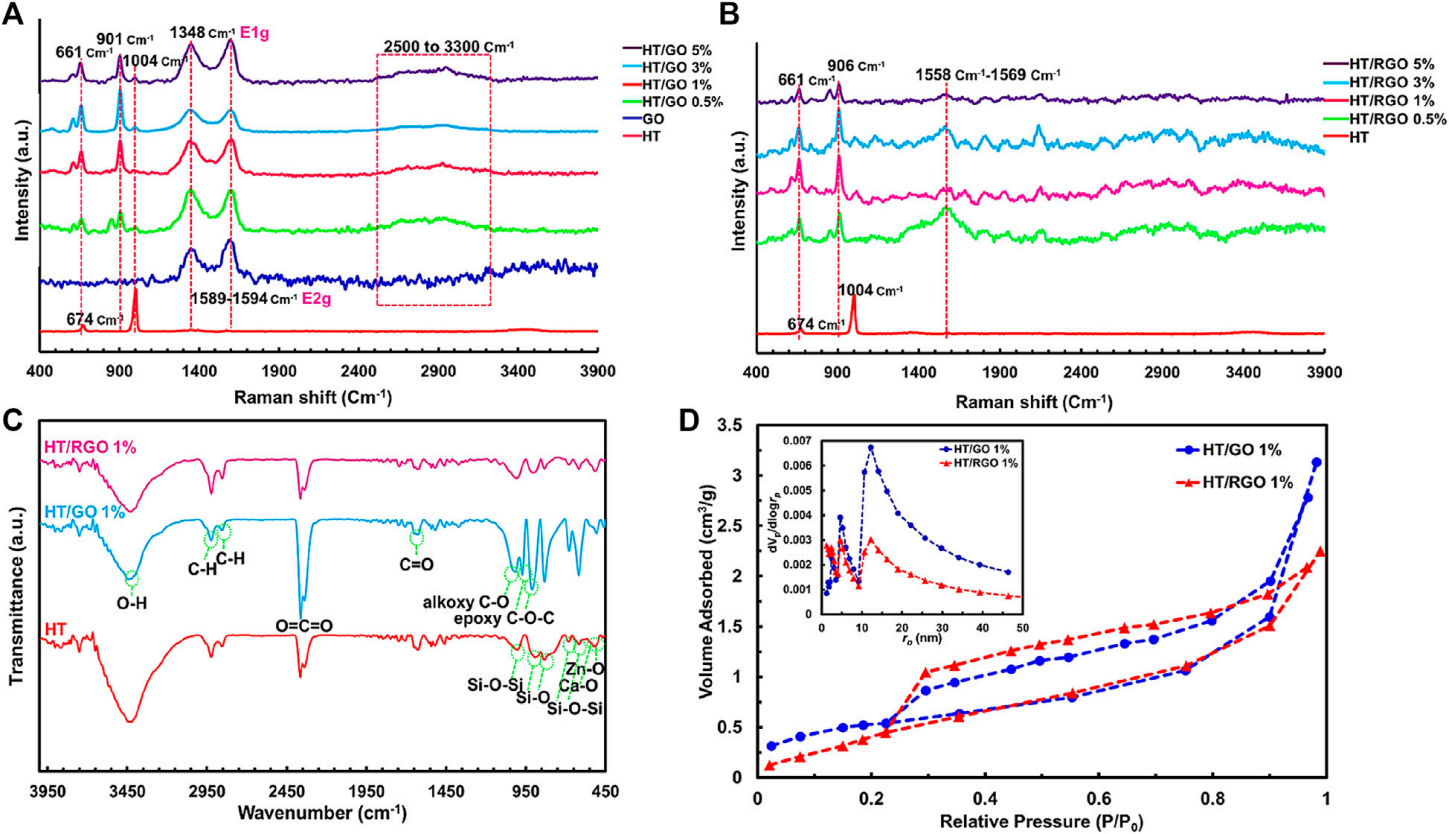
Raman images to characterize the electronic and structural properties of **(A)** HT/GO composite and **(B)** HT/RGO composite. **(C)** FTIR and **(D)** BET analyses of 1 % wt. HT/GO and 1 % wt. HT/RGO composites.

Raman analysis was also employed to characterize the electronic and structural properties of HT/GO composite including defect density, disorder and defect structures (Figures 4A, B). As can be seen in Figure 4A, in the Raman spectra of the pure HT, the peaks at 674 cm^−1^ and 1004 cm^−1^ are related to the symmetric bending and stretching vibrations of (Si-O-Si) and (SiO_3_) of the sorosilicate [Si_2_O_7_] structural group, respectively [38, 39, (Sharma et al., 1988; Contents Phys, 2012). Also, the lattice distortions of GO in HT/GO composites can be confirmed by Raman analysis (Jabbar et al., 2017). As seen, in the Raman spectrum of GO, the peak located at 1348 cm^−1^ corresponds to the vibrational mode E_1g_ of irregular carbons (sp^3^ carbon hybridization), which is called D-band (Figure 4A). Also, the peak observed at 1589-1594 cm^−1^ is related to the E_2g_ vibrational mode of the carbons in the graphene structure (sp^2^ carbon hybridization), which is called G-band (Bagherzadeh et al., 2016; Javidparvar et al., 2019). Also, the broad band in the range of 2500 cm^−1^ to 3300 cm^−1^, is called 2D (or G ′) band, which is related to the Zone-Boundary Phonon (Figure 4A). The intensity of this peak is related to the number of stacked graphene layers (Mehrjoo et al., 2015). As can be seen in Figure 3A, all peaks related to the structure of the pure HT and GO are observed in the Raman spectra of HT/GO composites confirming the co-existence of both structures in the composite. Also, it is observed that in GO composites, the peaks of HT are shifted toward lower wave numbers of 661 and 901 cm^−1^, while this significant shift was not observed for GO peaks in HT composites (Figure 4A). The blue shift of the HT peaks in the presence of GO percentages can be related to this fact that the capture of GO nanosheets in the HT structure increases the distance between the silicon-oxygen bonds, which leads to an increment of the bond distance and reducing the required energy to detect the bond. Moreover, 2D peak intensity for HT/GO composites is higher than that of pure GO sample, so that the maximum intensity of 2D peak was obtained by 1 wt. % GO, indicating a higher number of layers in this sample, which decreased with increasing GO content (Figure 4A). As known, the intensity I_D_/I_G_ ratio indicates the average size of the sp^2^ domains and the degree of disorder in graphene materials. The I_D_/I_G_ ratio for GO was found to be 0.82 which increased from 0.96 to 0.99 for HT/GO composites, as shown in Figure 4A, suggesting the presence of structural defects in GO lattice which goes along with size decrease of the sp^2^ domains (Mehrali et al., 2014). The Raman spectra of the HT/RGO tablets produced after the hydrothermal processes under 1375°C, exhibit significant changes compared to the spectra of pure samples (Figure 4B). As seen in Figure 4B, the peak of (SiO_3_) vibrations of HT composite remains intact after hydrothermal process and sintering compared to HT/GO, suggesting the successful incorporation of graphene layers between HT composite structures even under hydrothermal condition. In addition, exposure to high temperature during sintering causes the more shift of G band of graphene to a lower wave number of 1558–1569 cm^−1^ arising from the increased number of sp^2^ carbon atoms. Moreover, both D and 2D peaks are disappeared after sintering which indicates most oxygen containing functional groups C=C bonds in graphene oxide are reduced and removed during reduction at the high temperature (Figure 4B) (Jabbar et al., 2017; Bahtiar, 2018).

To further characterize of graphene layer incorporation into HT structure, the FT-IR spectra of free HT, and HT/GO 1% and the HT/RGO 1% composites were recorded and illustrated in Figure 4C. The FTIR spectrum of free HT shows two characteristic peaks at 516 and 526 cm^−1^ related to the Zn–O and Ca–O functional groups, and others that belong to the SiO_4_ groups including 616 and 686 cm^−1^ attributed to the Si−O−Si bending vibrations, and 832, 894 and 1008 cm^−1^ related to the symmetric stretching of Si−O and Si−O−Si. The HT also exhibited some peaks in the range of 1500-3500 cm^−1^ due to the exposure of powder to the atmosphere. For the HT/GO 1% composite, in addition to HT free absorption peaks, a number of peaks corresponding to the GO could be observed including 1630 and 2366 cm^−1^ resulting from stretching and bending vibrations of C=O, O–H and O=C=O groups, respectively, the symmetric stretching of CH_2_ at 2852 cm^−1^ and the asymmetric stretching of CH_2_ at 2924 cm^−1^, and a broad band about 3430 cm^−1^ which could be assigned to stretching vibration of O−H (Figure 4C). Moreover, the alkoxy carbonyl C-O bond at 1020 cm^−1^ and the epoxy group C-O-C at 970 and 910 cm^−1^ appear. These oxygen groups can be due to the conversion of graphite sp^2^ structure to the sp^3^ oxide regions in GO under oxidation, so that these epoxy and alkoxy carbonyl groups as well as the bending CO_2_ at 2366 cm^−1^ significantly decreased and the asymmetric and symmetric stretching of CH_2_ as the characteristic bonds of RGO nanosheets increased following hydrothermal reaction in the HT/RGO 1% composite (Figure 4C) (Mehrali et al., 2014; Mohammadi, 2015; Farzin et al., 2016; Shamsipur et al., 2019).

In final characterization step, N_2_ adsorption–desorption isotherms of HT/GO 1% and the HT/RGO 1% composites were investigated (Figure 4D) together with corresponding pore size distributions (inset). As seen, the adsorption-desorption isotherm type IV was observed, indicating the mesoporous structure for materials (Figure 4D). Really, hydrothermal condition inducing RGO formation did not change the mesoporous structure of graphene-based HT composite. The BET surface areas of the HT/GO 1% and HT/RGO 1% materials were almost the same (∼1.91 m^2^ g^−1^). In addition, the BET total pore volume and the mean pore diameter of HT/GO 1% were obtained 0.0048 cm^3^g^−1^ and 10.15 nm, respectively, which is higher than that of HT/GO 1% (0.0035 cm^3^ g^-1^, 7.26 nm). Moreover, from BJH method the single point adsorption total volume (V_P_) at P/P_0_ = 0.98 for the HT/GO 1% and the HT/RGO 1% materials were 0.0045 and 0.0037 cm^3^ g^−1^, respectively. These decreases of pore volume and pore diameter in HT/RGO could be due to the generation of the amount of defects in the mesoporous structure of HT composite after being scaffolded and hydrothermal treatment (Zhang, 2013; Karamian and Gheisari, 2015; Gharehdaghi et al., 2022).

### 3.2 *In Vitro* biocompatibility of HT/RGO composites

**FIGURE 5:**
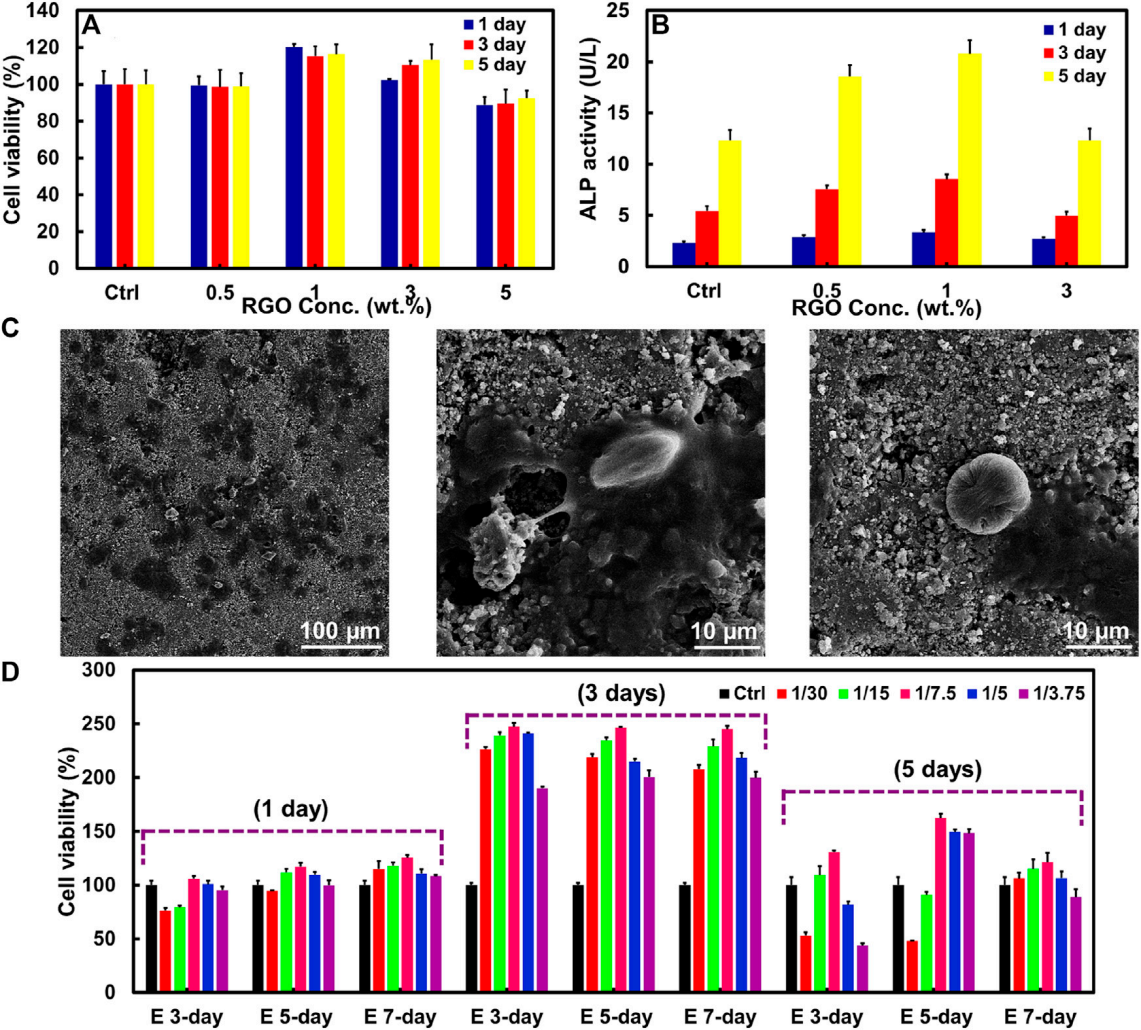
**(A)** MTT assay and **(B)** ALP activity of MC3T3-E1 cells on HT/RGO tablet scaffolds with different percentages. **(C)** SEM images of the adherent of MC3T3-E1 cells seeded on 1% wt. HT/RGO tablet scaffolds after 3 days. **(D)** MTT assay for the effect of various concentrations of E 3-day, E 5-day, and E 7-day samples prepared from 1 % wt. HT/RGO composite on cell proliferation of osteoblast human G-292.

The HT/RGO composite could be introduced a promising candidate for use in orthopedic hard tissue implants. So, the HT/RGO composite as an orthopedic implant should induce cellular adhesion, differentiation, and proliferation. In this way, the effect of RGO on the growth and proliferation of mouse osteoblastic (MC3T3-E1) cells was assessed qualitatively after 1, 3, and 5 days by MTT assay during which Osteoblasts were seed onto HT composite tablets containing different weight percentages of RGO (0.5, 1, 3, and 5 wt%). As depicted in Figure 5A, no cytotoxicity was found for the scaffold specimens and HT-1% wt. GO samples showed more cell viability than others without a significant difference between 1 and 5 days, probably due to the a higher number of RGO layers in composite structure as well as mesoporous HT structure as demonstrated by Raman and SEM analysis (Figures 2–4) providing higher surface area for cell adhesion (Mehrali et al., 2014; Abdollahi Boraei et al., 2021; Boraei et al., 2022).

To better knowledge of the RGO effect on the behavior of the MC3T3-E1 cells, osteoblast differentiation was evaluated using an ALP (alkaline phosphate activity) assay as an early marker in osteoblast differentiation for bone formation. Figure 5B exhibits the proliferation and ALP activity of the MC3T3-E1 cells cultured on pure HT for 5 days. As shown in Figure 5B, the maximum ALP activity was obtained for the cells cultured on HT/1 wt. % RGO composite and after that decreased for HT/3 wt. % RGO composite similar to the cells cultured on the pure HT as control. This result corresponds to the MTT assay. Moreover, the ALP activity of the cells significantly increased with RGO content in the HT composites. The ALP expression level of the HT−1 wt. % RGO composite was about 1.44–1.70 times higher than that of the pure HT ceramic, revealing its positive effect of developed 1 wt. % RGO composite on the differentiation of mouse osteoblast cells. The obtained ALP assay result of the developed HT−1 wt. % RGO composite is comparable (Jaiswal et al., 2013) and even more than other reported graphene-based scaffolds; for example, about two times more than that of HA/RGO and BSA-RGO/bredigite composites after 5 days (Liu et al., 2013a; Askari et al., 2021) and about four times than that of chitosan/GO (Hermenean et al., 2017) as well as about three times than akermanite bioceramics after 7 days (Xia et al., 2016).

To further confirm the attachment of cells on the surface of composite tablets, SEM technique was used to provide detailed images of cell morphology for the optimum composite state, HT/1 wt. % RGO. As can be seen in Figure 5C, the MC3T3-E1 cells are remarkably adhered on scaffolds with a typical elongation, healthy globular and flat shapes, and significant spreading in HT microstructures containing 1 wt. % RGO, suggesting normal cell growth and attachment processes. To further identify the efficacy of HT/RGO 1% material in bone tissue engineering field, the cellular proliferation was studied on the osteoblast human G-292 cells (Heydari et al., 2017; Askari et al., 2021) in various concentrations of HT/RGO 1% extracts (Figure 5D). Notably, by ICP analysis the concentrations of Ca ion were obtained 9.4 ppm for E 3-day and E 5-day samples and 21.3 ppm for E 7-day sample. Also, it was found about 0.56 ppm of Zn ion in all samples. It is clear that the cells proliferated significantly from day 1 to day 3 and after 5 days the proliferation rate decreases to some extent. However, osteoblastic cell proliferation shows more than 2 times after 3 days and 1.5 times after 5 days in comparison with 1 day. According to the results, the maximum proliferation rate was obtained for 1/7.5 dilution of 5-day extract sample (E 5-day). More importantly, the cell viability of E 5-day with 1/7.5 dilution reached from 117% to 245% after 3 days and then to 165% after 5 days (Figure 5D). This optimum state (245% at day 3) could be an outstanding result compared to some other previously reported studies such as the akermanite bioceramics, Mg-doped HA, and aligned fibroporous poly (carbonate urethane)/GO (Mehrjoo et al., 2015; Thampi et al., 2015; Xia et al., 2016). Based on the MTT analysis, the 1/7.5 dilution of HT/RGO 1% 5-day extract (E 5-day) could be chosen as the appropriate concentration for the future studies. It is noted that the surgical implantation in appropriate animal model and the related immune tolerance tests by our developed composite together with other completing tests such as real-time quantitative RT-PCR for the mRNA expression of cells will be presented in our next study.

Overall, the proliferation, cell attachment, and differentiation data for pure HT and HT/RGO composites demonstrate that the biocompatible HT/1 wt. % RGO composite has a high degree of composite−osteoblast interaction and cell adhesion in which the use of 1 wt. % of RGO can significantly enhance *in vitro* bone formation ability. Really, the findings of the current study indicate that GO has adequate biocompatibility for using as a biomaterial, and adding GO into the HT matrix significantly improves the cellular response to the HT nanocomposite and thus the favorable structure properties of graphene-based HT with high degree of porosity and surface area, stability, cell viability and ALP activity can create a bioplatform for the possibility of applying in the osteoporosis, load-bearing hard tissue implants and other bone tissue engineering scaffolds.

### 3.3 Mechanical properties of HT/RGO composites

**FIGURE 6:**
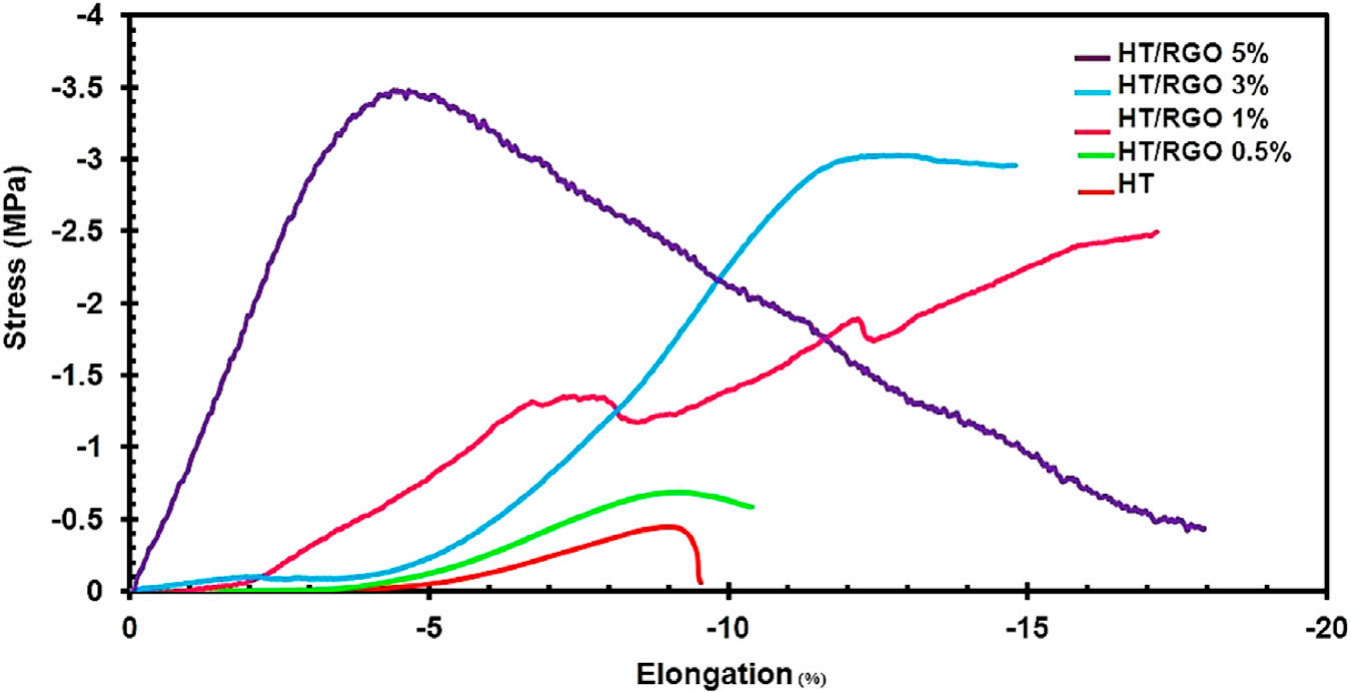
The compressive stress-strain curves of HA/RGO nanocomposites.

Mechanical properties of scaffolds is one of the important parameters to be considered in tissue engineering field (Vedadghavami et al., 2017). In this way, the compressive stress-strain curve of HT/RGO composites was recorded and shown in Figure 6. Moreover, the obtained mechanical parameters are reported in Table 1. According to the previous studies (Ghomi et al., 2016), in the case of porous scaffolds, there are three regions which could be obtained from the stress–strain test: 1) a linear region which is almost directly related to the strain and continues until the final compressive strength was achieved; 2) Failure of the under-pressure; 3) composite compression and pore closure by increasing the pressure. As shown in Figure 6, these three areas can be seen in the corresponding diagrams. As seen, the ultimate compressive strength of the samples increased with increasing the content of RGO with values of 0.42, 0.68, 2.49, 3.00 and 3.43 MPa for the samples containing 0, 0.5, 1, 3 and 5 wt. % RGO, respectively. The obtained results are completely in the compressive strength range of sponge bone (0.2–4 MPa) which prevent the shield stress phenomenon (Magness et al., 2017). These results indicate the high capability of the developed HT/RGO composites for use in bone tissue engineering. In fact, the reduced graphene oxide nanosheets acts as a filler and flux in the HT composite, allowing the reduction of sintering temperature and consequently improving the mechanical properties of the studied composites (Magness et al., 2017). The area below the stress-strain curves increased with increasing the amount of the RGO, indicating a higher toughness of the HT composite with the higher amounts of RGO. The obtained results indicates that in the presence of 5 wt. % of the RGO, the toughness value increased by about 11.8 times more than the value obtained for the sample without RGO. In addition, according to Table 1, it is clear that the compressive modulus value of the nano- and microcomposite increased with increasing the content of RGO in the HT-based composite, so that the maximum value was obtained for 5 wt. % of RGO sample with about 86% higher compression modulus value than the free RGO sample. Moreover, the highest value of maximum strain and toughness parameters belonged to the HT-5 wt. % RGO. This indicates that the optimal sample in terms of mechanical properties could be the sample with 5wt. % RGO.

**TABLE 1:** The obtained mechanical parameters from the compressive test for HA/RGO nanocomposites.

Sample	Ultimate compressive strength (MPa	Compressive modulus (MPa)	Maximum strain (%)	Toughness (J/m^2^)
Hardystonite	0.42	0.14	9.52	2.89
0.5% RGO	0.68	0.16	10.35	3.36
1% RGO	2.49	0.28	17.14	17.54
3% RGO	3.00	0.61	14.75	19.56
5% RGO	3.43	0.98	17.94	34.33

Another important mechanical property for the proposed composites is their bending properties, which are obtained by a three-point bending test (Table 2). The related results are reported in Table 2. According to Table 2, it is clear that the mechanical parameters obtained from the bending test increased with increasing the amount of RGO incorporated in the HT-based composite as obtained from the compressive test. According to these results, it can be seen that the bending strength and bending modulus values increased respectively about 24.7% and 35.8% in the nanocomposite containing 5 %wt. RGO compared to the composite without RGO. In fact, due to the high aspect ratio of the reduced graphene oxide nanosheets and the high interface of this additive with the HT matrix, this reinforcing agent dispersed well in the matrix and fills the pores and cavities of the composite. Due to the susceptibility of these cavities to nucleation and the growth of cracks due to the stress concentration at these regions, the nanosheets block or divert the growth path of the cracks by filling the composite cavities, and so improve the mechanical properties of the produced nanocomposite (Javidparvar et al., 2020).

**TABLE 2:** The obtained mechanical parameters from the bending test for HA/RGO nanocomposites.

Sample	Bending strength (MPa)	Bending modulus (GPa)
Hardystonite	20.77	5.89
0.5% RGO	21.08	6.12
1% RGO	23.83	7.43
3% RGO	24.32	7.99
5% RGO	27.59	9.18

For TOC, only.

## 4 Conclusion

In summary, we used a sol-gel method followed by hydrothermal processing method to synthesize hardystonite (HT phase)-reduced graphene oxide composite powders. This method produced HT nano-/microstructures with sizes up to 200 nm accompanied by some nanowires in the HT phase containing 0.5-1 wt. % GO with lengths of several micrometers. After hydrothermal process at 1375°C, the presence of RGO together with an increased porosity in the composite was demonstrated using Raman and SEM analysis. Moreover, it was found that the addition of GO into HT pure sample, did not significantly affect the crystallinity of the resulting particles in HT/GO composite tablets. Interestingly, the HT−1 wt. % RGO composite induced the effective proliferation of osteoblastic cells and significantly increased the ALP expression level on MC3T3-E1 cells with time compared to the pure HT ceramics. Moreover, the developed HT/RGO composite has successfully demonstrated a highly improvement in mechanical performance with increasing RGO content compared with the pure HT composite. Altogether, our results suggest that integrating 1 wt. % of GO into the HT resulted in a promising composite material which could be considered as a bone implant candidate with improved biological and mechanical properties.

## Data Availability

The raw data supporting the conclusions of this article will be made available by the authors, without undue reservation.
